# INTERROGATING AGEISM

**DOI:** 10.1093/geroni/igad104.1989

**Published:** 2023-12-21

**Authors:** Douglas Hanes

**Affiliations:** Stony Brook University, Stony Brook, New York, United States

## Abstract

This paper identifies and addresses a puzzle within the conceptualization of ageism. According to the Reframing Aging Initiative, “Ageism refers to stereotypes, prejudice, and discrimination directed toward people on the basis of age”; these adversely affect older people’s lives in daily lives, including in employment and healthcare. Yet, unlike other marginalized groups, like people of color and women, older people are not underrepresented in powerful positions nor are they worse off. Indeed, they tend to fare better than the population overall: the mean age of US CEOs is 59 years; US Representatives and Senators are 58.4 and 64.3, respectively. Older Americans are more likely to be homeowners and less likely to be unemployed or food-insecure than the overall population. This paper considers several possible explanations. First, unlike racism or sexism, ageism may not be structural, but personal: present in individuals’ attitudes and interactions, but without population-level effects, codification, or institutionalization. Second, ageism may only hold intersectionally, becoming operational under conditions of structural oppression like racism and sexism. For example, while older US whites have less risk of food insecurity than younger whites, older Black Americans are at greater risk than younger Blacks. Finally, ageism may be mitigated by selective mortality: while older people face ageism daily, not everyone reaches older age. Wealthier people and whites are more likely to live to older age; older people’s greater power and welfare reflect their earlier status. Each option presents benefits and drawbacks; together they allow us to address those whom ageism most harms.

